# Generation of a bile salt export pump deficiency model using patient-specific induced pluripotent stem cell-derived hepatocyte-like cells

**DOI:** 10.1038/srep41806

**Published:** 2017-02-02

**Authors:** Kazuo Imagawa, Kazuo Takayama, Shigemi Isoyama, Ken Tanikawa, Masato Shinkai, Kazuo Harada, Masashi Tachibana, Fuminori Sakurai, Emiko Noguchi, Kazumasa Hirata, Masayoshi Kage, Kenji Kawabata, Ryo Sumazaki, Hiroyuki Mizuguchi

**Affiliations:** 1 Department of Child Health, Faculty of Medicine, University of Tsukuba, Ibaraki, Japan; 2 Laboratory of Biochemistry and Molecular Biology, Graduate School of Pharmaceutical Sciences, Osaka University, Osaka, Japan; 3 Laboratory of Hepatocyte Regulation, National Institutes of Biomedical Innovation, Health and Nutrition, Osaka, Japan; 4 The Keihanshin Consortium for Fostering the Next Generation of Global Leaders in Research (K-CONNEX), Kyoto University, Kyoto, Japan; 5 Department of Diagnostic Pathology, Kurume University Hospital, Fukuoka, Japan; 6 Department of Surgery, Kanagawa Children’s Medical Center, Kanagawa, Japan; 7 Laboratory of Applied Environmental Biology, Graduate School of Pharmaceutical Sciences, Osaka University, Osaka, Japan; 8 Laboratory of Regulatory Sciences for Oligonucleotide Therapeutics, Clinical Drug Development Project, Graduate School of Pharmaceutical Sciences, Osaka University, Osaka, Japan; 9 Department of Medical Genetics, Faculty of Medicine, University of Tsukuba, Ibaraki, Japan; 10 Laboratory of Stem Cell Regulation, National Institutes of Biomedical Innovation, Health and Nutrition, Osaka, Japan; 11 Laboratory of Biomedical Innovation, Graduate School of Pharmaceutical Sciences, Osaka University, Osaka, Japan; 12 Global Center for Medical Engineering and Informatics, Osaka University, Osaka, Japan

## Abstract

Bile salt export pump (BSEP) plays an important role in hepatic secretion of bile acids and its deficiency results in severe cholestasis and liver failure. Mutation of the *ABCB11* gene encoding BSEP induces BSEP deficiency and progressive familial intrahepatic cholestasis type 2 (PFIC2). Because liver transplantation remains standard treatment for PFIC2, the development of a novel therapeutic option is desired. However, a well reproducible model, which is essential for the new drug development for PFIC2, has not been established. Therefore, we attempted to establish a PFIC2 model by using iPSC technology. Human iPSCs were generated from patients with BSEP-deficiency (BD-iPSC), and were differentiated into hepatocyte-like cells (HLCs). In the BD-iPSC derived HLCs (BD-HLCs), BSEP was not expressed on the cell surface and the biliary excretion capacity was significantly impaired. We also identified a novel mutation in the 5′-untranslated region of the *ABCB11* gene that led to aberrant RNA splicing in BD-HLCs. Furthermore, to evaluate the drug efficacy, BD-HLCs were treated with 4-phenylbutyrate (4PBA). The membrane BSEP expression level and the biliary excretion capacity in BD-HLCs were rescued by 4PBA treatment. In summary, we succeeded in establishing a PFIC2 model, which may be useful for its pathophysiological analysis and drug development.

Bile salt export pump (BSEP) is a key molecule for the generation of bile flow in humans^1^. BSEP deficiency causes severe intrahepatic cholestasis and liver failure. Progressive familial intrahepatic cholestasis (PFIC) is one of the cholestatic diseases in children, and PFIC type 2 (PFIC2) is a form of infantile cholestatic disorder that occurs despite normal serum levels of gamma glutamyl transferase (GGT)^2^. PFIC2 is caused by mutations in the *ABCB11* gene that encodes BSEP^3^. Certain mutations cause deficiency in the membrane expression of BSEP owing to accelerated proteasome-mediated degradation^4^,^5^,^6^,^7^. The main clinical features of BSEP-deficiency are severe jaundice, pruritus, and intrahepatic cholestasis, followed by liver failure and juvenile hepatobiliary carcinoma^2^,^8^,^9^. While orthotopic liver transplantation (OLT) is the major curative approach for BSEP-deficiency^2^,^10^, some BSEP-deficient patients treated with OLT experience a relapse of intrahepatic cholestasis due to the presence of autoimmune antibodies against BSEP^11^,^12^. Therefore, it is important to elucidate the pathophysiology of PFIC2 in order to develop novel therapies for its treatment.

Knockout mouse models are often used for the elucidation of disease mechanisms. However, *Bsep/Abcb11*-knockout mice are not a valid model for reproducing PFIC2 phenotypes because of species differences in the functions of hepatic transporters^13^. Specifically, multiple drug resistance 1 (Mdr1) compensates for the biliary excretion functions of Bsep in mice, whereas no transporter compensates for these functions in humans^14^. To analyze the functions of mutated human *ABCB11*, BSEP-overexpressing cell lines are used widely. BSEP-overexpressing cells are useful for analyzing bile acid kinetics mediated by mutated BSEP, but not for analyzing the function of BSEP having mutations in the non-coding region of the *ABCB11* or other genes. In fact, one-third of patients with PFIC harboring normal GGT do not have mutations in *ABCB11* or *ATP8B1* genes^15^,^16^. In addition, some *ABCB11* mutations result in disruption of pre-mRNA splicing^17^. To perform pathological analysis of these mutations, primary human hepatocytes should be obtained from patients^18^. Therefore, a novel *in vitro* disease model containing the whole genome information of patients with BSEP-deficiency is necessary to further elucidate the disease mechanisms and BSEP regulation.

Human induced pluripotent stem cells (iPSCs) can be obtained by reprogramming somatic cells^19^. Patient-specific iPSCs and their derivatives are expected to offer novel disease models^20^. Moreover, the iPSC technology has already shown great potential for the discovery of new therapies against several diseases^21^. The utilization of patient-specific iPSCs and their derivatives would be advantageous for generating a disease model in humans, because the phenotype can be evaluated without consideration of species differences. In this study, human iPS cell-derived hepatocyte-like cells (HLCs) were generated from patients with BSEP-deficiency. As we have already established a highly efficient hepatocyte differentiation protocol, an almost homogenous hepatocyte population (more than 80%) could be generated from human iPS cells independently of iPS cell lines^22^,^23^. In this study, human iPSC lines were established from two patients with BSEP-deficiency (BD-iPSC), and then differentiated into the HLCs. Finally, we examined whether the BD-iPSC derived HLCs (BD-HLCs) recapitulated the pathophysiology of PFIC2, specifically the aberrant splicing of *ABCB11* mRNA, reduction of membrane BSEP expression and impairment of biliary excretion capacity.

## Materials and Methods

### Ethical statement

This study was approved by the ethics committees of the University of Tsukuba and the National Institutes of Biomedical Innovation, Health and Nutrition. All experiments were performed in accordance with relevant guidelines and regulations, and with the approval of the University of Tsukuba and the National Institutes of Biomedical Innovation, Health and Nutrition. Written informed consent was obtained from the participants or their parent.

### Generation of human iPSCs from peripheral blood mononuclear cells

Peripheral blood mononuclear cells (PBMCs) were separated from whole blood using Ficoll gradient separation, and then cultured with plate-bound anti-CD3 monoclonal antibody (Becton, Dickinson and Company) and X-VIVO10 medium (Lonza) containing 10 ng/mL recombinant IL-2 (Thermo fisher scientific). To generate human iPSCs from PBMCs, Yamanaka four factor-expressing Sendai-virus (SeV) vectors (CytoTune-iPS For Blood Cells; DNAVEC) was used^24^. Twenty-four hours after plating (1.87 × 10^5^ cells/cm^2^), the PBMCs were transduced with the SeV vector at a multiplicity of infection (MOI) of 20. Twenty-four hours after the transduction, the medium was replaced with fresh X-VIVO 10 medium. The next day, the SeV-transduced PBMCs were plated onto a mitomycin C-treated EmbryoMax Primary Mouse Embryo Fibroblasts (MEF; Merck Millipore) feeder layer. The PBMCs were cultured with ReproStem (ReproCELL) medium containing 5 ng/mL fibroblast growth factor 2 (FGF2; Katayama Chemical Industries) for 2–3 weeks. The SeV genome cDNA synthesized from RNA of human iPSCs was amplified by RT-PCR using Ex Taq DNA polymerase (TaKaRa Bio Inc.). The PCR products were separated by electrophoresis on 2% agarose gels and visualized by staining with ethidium bromide. The SeV RNA genome was not detected in human iPSCs passaged more than 10 times (Figure S1D). The PCR primer sequences used in this study are described in Table S1.

### Human ESCs/iPSCs Culture

Human embryonic stem cell (ESC) line, H9 (WiCell Research Institute), was maintained on MEF feeder layer with ReproStem medium supplemented with 5 ng/mL FGF2. Human ESCs were used following the Guidelines for Derivation and Utilization of Human Embryonic Stem Cells of the Ministry of Education, Culture, Sports, Science and Technology of Japan and furthermore, and the study was approved by Independent Ethics Committee. Human iPSC lines were maintained on MEF feeder layer with ReproStem medium supplemented with 10 ng/mL FGF2.

### Primary Human Hepatocyte Culture

Platable cryopreserved human hepatocytes were purchased from VERITAS (lot YOW). The vials of hepatocytes were rapidly thawed in a shaking water bath at 37 °C, and then the contents of the vial were emptied into prewarmed Cryopreserved Hepatocyte Recovery Medium (CHRM, Life Technologies) and the suspension was centrifuged at 750 rpm for 10 min at room temperature. The hepatocytes were seeded at 1.25 × 10^5^ cells/cm^2^ in HCM containing 10% FBS (Life Technologies) onto Cellmatrix Type I-A acid-soluble type I collagen (Nitta Gelatin)-coated plates. The medium was replaced at 6 hr after seeding. The hepatocytes, which were cultured 48 hr after plating the cells, were used in the experiments.

### Hepatocyte differentiation *in vitro*

For hepatocyte differentiation from human ESC and iPSC lines, the differentiated cells were constantly removed by manually picking them up. Before the initiation of hepatocyte differentiation, human ESCs/iPSCs were dissociated into clumps by using dispase (Roche Diagnostics) and plated onto BD Matrigel Basement Membrane Matrix Growth Factor Reduced (Becton, Dickinson and Company). These cells were cultured in the mouse embryo fibroblasts-conditioned medium for 3–4 days. The differentiation protocol for the induction of definitive endoderm cells, hepatoblast-like cells, and hepatocyte-like cells was based on our previous reports with some modifications^22^,^23^. Briefly, in the definitive endoderm differentiation, human ESCs/iPSCs were cultured with the L-Wnt3A-expressing cell (CRL2647; ATCC)-conditioned RPMI1640 medium (Sigma) containing 100 ng/mL Activin A (R&D Systems), 1% GlutaMAX (Thermo fisher scientific), 0.2% fetal bovine serum (FBS; Thermo fisher scientific), and 1 × B27 Supplement Minus Vitamin A (Thermo fisher scientific) for 4 days. For the induction of hepatoblasts, the definitive endoderm cells were cultured with RPMI1640 medium containing 30 ng/mL bone morphogenetic protein 4 (BMP4) (R&D Systems) and 20 ng/mL fibroblast growth factor 4 (R&D Systems), 1% GlutaMAX, and 1 × B27 Supplement Minus Vitamin A for 5 days. To perform the hepatocyte differentiation, the hepatoblasts were cultured with RPMI1640 medium containing 20 ng/mL hepatocyte growth factor (HGF) (R&D Systems), 1% GlutaMAX, and 1 × B27 Supplement Minus Vitamin A for 5 days. Finally, the cells were cultured with hepatic maturation medium (hepatic maturation medium consists of Hepatocyte Culture Medium (HCM; Lonza, without epidermal growth factor (EGF)) containing 20 ng/mL oncostatin M (OsM) and 3% GlutaMAX) for 6 days. On day 20, the Matrigel solution (diluted by the hepatic maturation medium; the final Matrigel concentration was 0.25 mg/mL) was overlaid on the cells. The next day, the medium was changed with the hepatic maturation medium, and then the cells were cultured until day 25.

### DNA sequence analysis

The exonic sequences of the *ABCB11* gene were analyzed in the PBMCs/iPSCs of a control and patients with BSEP deficiency by direct sequencing of PCR products. The DNA was extracted from the PBMCs/iPSCs of a control and a patient with BD using a DNeasy Blood & Tissue Kit (Qiagen). The cDNA sequence of the *ABCB11* gene was also analyzed by the direct sequencing of PCR products. The cDNA was synthesized from RNA of the Control, BD1 and BD2-HLCs. The PCR products were gel-purified, extracted with the GENECLEAN kit (MP Biomedical) according to the manufacturer’s instructions, and used as templates for the sequencing reaction with the Big Dye Terminator kit v3.1 (Applied Biosystems). Sequence analysis was performed by a Genetic Analyzer 3130 (Applied Biosystems). The sequences were compared to the reference sequence (GenBank accession number NM_003742.2). The nucleotide numbering was relative to the first adenine (+1) of initiation ATG codon. The PCR primer sequences used in this study are described in Table S2.

### Biliary excretion index assay

To evaluate the biliary excretion capacity *in vitro*, the biliary excretion index (BEI) was calculated^25^,^26^. The Control, BD1, and BD2-HLCs on day 25 of culture were prepared, and then the cells were washed with HBSS buffer three times. Ten minutes after the cells were incubated with HBSS buffer or calcium-free HBSS buffer, the buffer was exchanged for HBSS buffer containing 5 μm cholyl-lysyl- fluorescein (CLF), and the cells were incubated for 10 minutes. Uptake of CLF was stopped by adding cold HBSS buffer. The cells were washed with HBSS buffer, and lysed by 1% Triton X-100. The amount of CLF in the lysate was estimated by measuring the fluorescence at 492 nm and 536 nm by using a microplate reader (Genios, Tecan). BEI was calculated as follows. BEI = 100*(HBSS-HBSS(Calcium free))/HBSS%

## Results

### Patients with BSEP-deficiency

**Figure 1:**
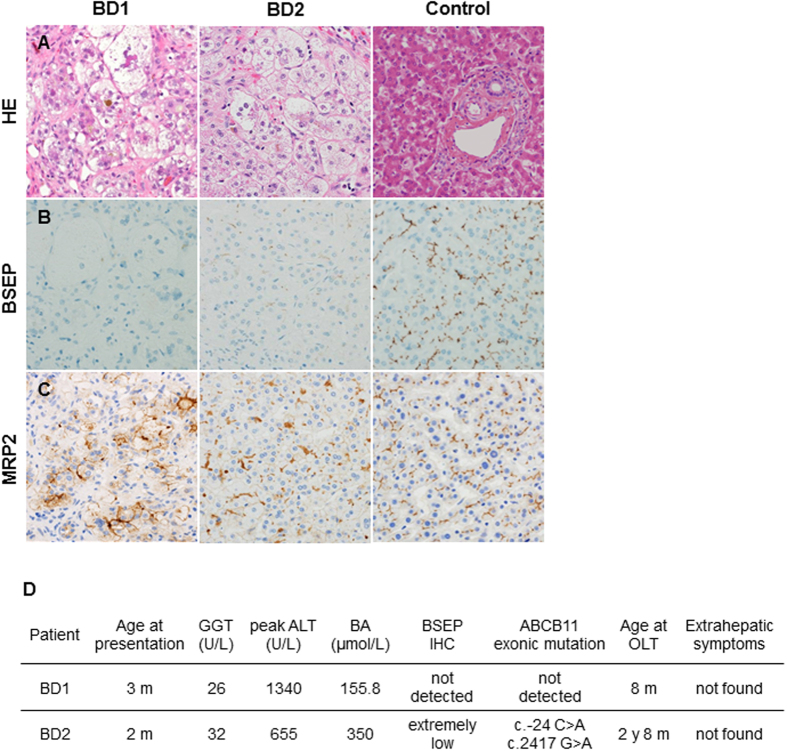
Pathological examination in liver sections. (**A**) Staining of liver sections with hematoxylin and eosin. Immunostaining analyses of liver sections with BSEP (**B**) and MRP2 (**C**) (original magnification: 400×). (**D**) Characteristics of both the BSEP-deficient patients.

A BSEP-deficient patient, named as BD1, was a 6-year-old girl. At the age of 3 months, she experienced severe jaundice and cholestasis with normal serum GGT levels. In addition, liver histological findings showed cholestasis, giant cell transformation, hepatocellular swelling (Fig. 1A, left), and negative BSEP immunostaining (Fig. 1B, left). At 8 months of age, due to liver failure, a successful OLT was performed. The clinical course since OLT has been good, and currently she does not have any hepatic or extrahepatic symptoms. She was diagnosed as having progressive cholestasis with normal-GGT and BSEP deficiency but there were no mutations in the exons of the *ABCB11* gene. The other BSEP-deficient patient, named as BD2, was a 13-year-old boy. He suffered from severe jaundice and cholestasis with normal serum GGT at the age of 2 months. Gene analysis of peripheral blood cells showed novel compound heterozygous mutations in the *ABCB11* gene, c.−24C > A (5′-UTR; five prime untranslated region) and c.2417G > A (p.G806D). Trio analysis identified that these *ABCB11* mutations were transmitted from the patient’s parents. Liver histological findings showed evidence for PFIC2; giant cell transformation, hepatocellular swelling, and cholestasis (Fig. 1A, middle). Consistently, liver immunohistochemistry indicated extremely low canalicular BSEP expression compared to control liver (Fig. 1B, middle). The other canalicular transporter, MRP2 was expressed in the BD2 patient’s liver to a degree similar to the control liver (Fig. 1C, middle). These findings were compatible with the typical symptoms of PFIC2. At 2 years of age, he underwent a successful liver transplantation from his mother. Clinical course since the procedure has been good, and the patient currently has no symptoms. Detailed information about these patients with BSEP-deficiency is shown in Fig. 1D.

### Generation of patient-specific iPSCs

An overview of human iPSC generation is shown in Figure S1A. Two patients with BSEP-deficiency (BD1 and BD2) were included in this study. To generate human iPSCs from these subjects, blood cells were collected, and the separated peripheral blood mononuclear cells (PBMCs) were transduced with Yamanaka four factor-expressing Sendai viral (SeV) vectors^24^. Twenty days after the SeV transduction, BD1 and BD2-iPSC colonies were obtained. Control-iPSCs had been generated previously from the peripheral blood cells of a healthy female^27^.

DNA sequence analyses showed that there were no differences in *ABCB11* exons between the iPSCs and their parent PBMCs. This meant that the *ABCB11* exonic mutations in BD2-iPSCs were consistently inherited from the parental PBMCs. Next, to characterize the human iPSC lines, immunostaining and real-time RT-PCR analyses of pluripotent markers were performed. Human iPSCs as well as ESCs (H9) were positive for OCT4, a pluripotency marker (Fig. S1B). The gene expression levels of the pluripotent markers (*OCT4* and *NANOG*) in the iPSCs were higher than those in the PBMCs, and similar to those in human ESCs (Fig. S1C). We confirmed that the SeV RNA genome was not detected in human iPSCs (Fig. S1D). To examine whether the iPSCs have the ability to differentiate into cells of the three germ layers, embryoid bodies (EBs) were generated. Human iPSC-derived EBs were positive for the endodermal marker (AFP), mesodermal marker (cTnT), and ectodermal marker (Nestin) (Fig. S1E). The gene expression levels of the endodermal markers (*AFP* and *TTR*), mesodermal markers (*NKX2.5* and *BRACHYURY)*, and ectodermal markers (*NEUROD1* and *PAX6*) were higher in iPSC-derived EBs than in undifferentiated cells (Fig. S1F). Taken together, these results indicated that human iPSCs were successfully generated from patients with BSEP-deficiency.

### Hepatocyte differentiation from human iPSCs

**Figure 2:**
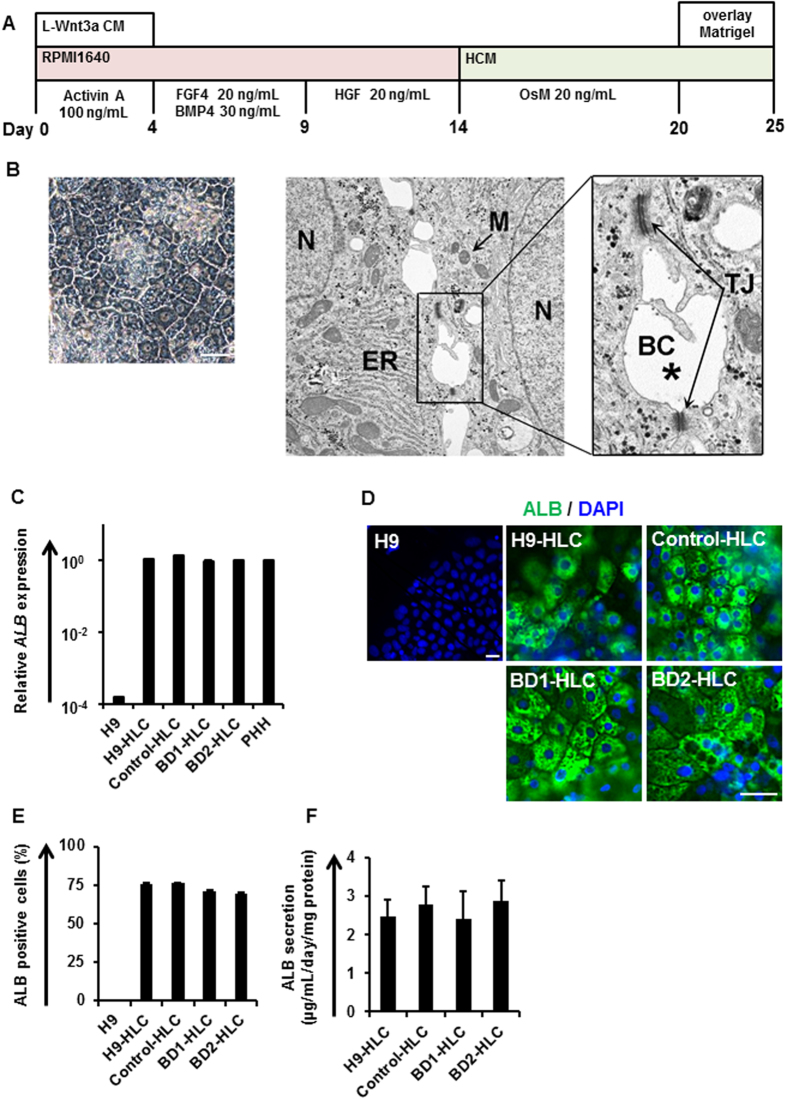
Differentiation of human iPSCs into hepatocyte-like cells. (**A**) The protocol for hepatocyte differentiation from human ESCs (H9) and human iPSCs. (**B**) Left panel is a phase contrast image of Control-HLCs at day 25. Middle panel is a transmission electron microscopy image of Control-HLCs (original magnification: 9300×). Right panel shows a further magnified image. Black asterisk indicates the canalicular space. BC, bile canaliculus; TJ, tight junction; ER, endoplasmic reticulum; M, mitochondria; N, nucleus. (**C**) Gene expression levels of *ALB* in HLCs derived from H9 cells or human iPSCs as examined by real-time RT-PCR. The gene expression level in PHH was taken as 1.0. Data represent mean ± SD (*n* = 3). (**D**) Immunostaining analyses of ALB in the HLCs derived from H9 cells or human iPSCs. Nuclei were counterstained with DAPI. (**E**) Percentage of ALB-positive cells in HLCs derived from H9 cells or iPSCs, as measured by flow cytometry. Data represent mean ± SD (*n* = 3). (**F**) ALB secretion levels of HLCs derived from H9 cells or human iPSCs as examined by ELISA. Data represent mean ± SD (*n* = 8). Scale bars represent 20 μm.

The protocol for hepatocyte differentiation was based on our previous report with some modifications (Fig. 2A)^22^,^23^. HLCs were overlaid with Matrigel on day 20 of hepatic cell differentiation, to promote the formation of bile canaliculi; the morphology was similar to that of human hepatocytes^28^. Moreover, transmission electron microscopy showed biliary canaliculi and tight junction structures in the HLCs (Fig. 2B). To investigate whether the biliary canaliculi were constructed between the HLCs, a bile secretion assay was performed using cholyl-lysyl-fluorescein (CLF), a fluorescent bile acid analog. CLF accumulation was detected in bile canaliculi of the Control-iPSC derived HLCs (Control-HLCs) overlaid with Matrigel (Fig. S2). To estimate the hepatocyte differentiation capacity of the iPSCs, albumin (ALB) expression levels were examined by real-time RT-PCR, immunostaining, flow cytometry and enzyme-linked immuno-sorbent assay (ELISA). The *ALB* gene expression levels in the iPSC-HLCs were similar to those in primary human hepatocytes (PHH) plated for 48 h (Fig. 2C). The HLCs stained positively for ALB (Fig. 2D), and the percentage of ALB-positive cells was more than 70% (Fig. 2E). In contrast, undifferentiated H9 cells were negative for ALB (Fig. 2D). The HLCs could abundantly secrete ALB into the culture medium (Fig. 2F). These results demonstrate that both efficient hepatocyte differentiation and comparative analysis of the HLCs could be achieved using our differentiation protocol.

### Sequencing analysis of *ABCB11* mRNA in the HLCs

**Figure 3:**
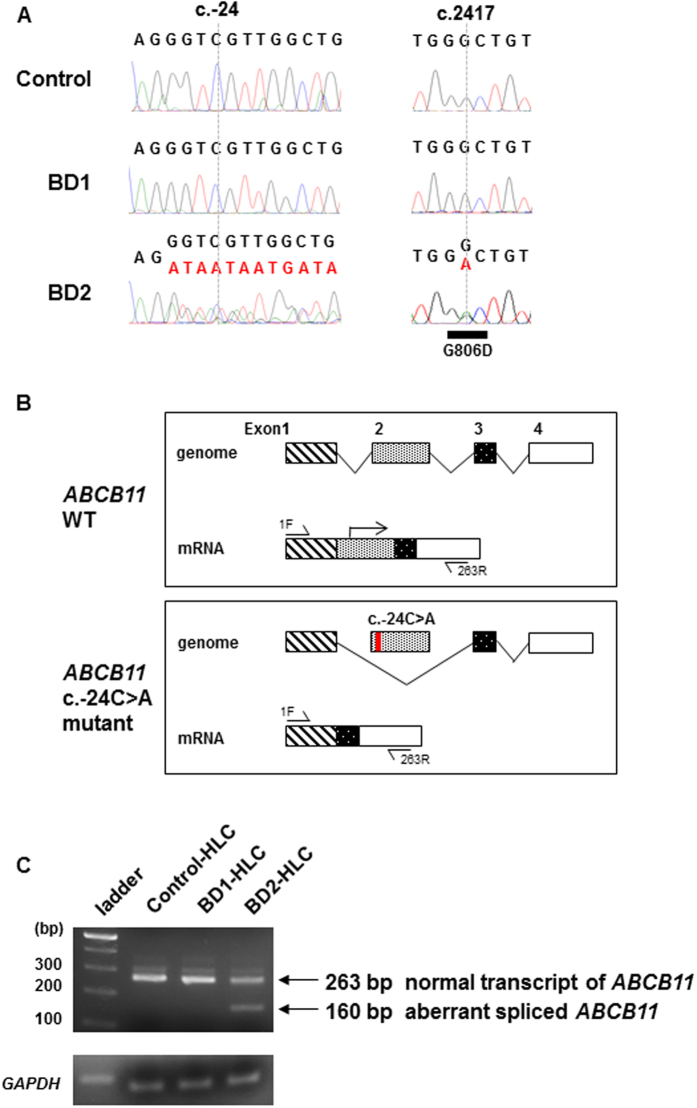
Sequence analysis of *ABCB11* mRNA using RNA extracted from the HLCs. (**A**) Sequence analysis of *ABCB11* mRNA using RNA extracted from the Control, BD1, and BD2-HLCs. (**B**) A schematic diagram of wild type (WT) and mutant form (BD2 patient) of *ABCB11* mRNA. (**C**) RT-PCR of normal and aberrantly spliced mRNA products of *ABCB11* in the different HLCs.

Up to now, a mutation in the 5′-UTR of the *ABCB11* gene has not been reported in BSEP-deficiency^2^,^10^. To examine whether the *ABCB11* mutation in the 5′-UTR mutation (c.-24C > A) would cause aberrant splicing in the HLCs derived from BD2-iPSCs (BD2-HLCs), direct sequence analysis of *ABCB11* cDNA was performed. Exon 2 sequence of *ABCB11* cDNA was heterozygously eliminated in BD2-HLCs (Fig. 3A). This suggested aberrant splicing of *ABCB11* in BD2-HLCs. A schematic diagram of the predicted *ABCB11* mRNA is presented in Fig. 3B (upper panel). In BD2-HLCs, aberrant splicing resulted in elimination of the proper initiation codon in *ABCB11* (Fig. 3B, lower panel, the red mark indicates the 5′-UTR mutation). These results suggested that the mutation in the 5′-UTR of the *ABCB11* gene (c.-24C > A) prevented normal BSEP translation. To investigate the aberrant splicing forms, RT-PCR analysis for *ABCB11* exons was performed, using primers (1F and 263R, Table S1) amplifying the region indicated in Fig. 3B. Agarose gel electrophoresis of the PCR product identified a smaller band of 160 bp in the BD2-HLC lane (Fig. 3C). The size of this PCR product was consistent with the size of aberrantly spliced form of *ABCB11* containing exons 1, 3, and 4. A heterozygous missense *ABCB11* gene mutation (c.2417G > A, p.G806D) was also observed in the transcripts of BD2-HLCs (Fig. 3A). The pathogenicity of this novel missense mutation was predicted by Polyphen-2 (Polymorphism Phenotyping V2, http://genetics.bwh.harvard.edu/pph2/) and SIFT (Sorting Intolerance From Tolerant, http://sift.jcvi.org/) tools, and its effect is anticipated to be damaging (Table S3).

### Recapitulation of the PFIC2 phenotype using BD-HLCs

**Figure 4:**
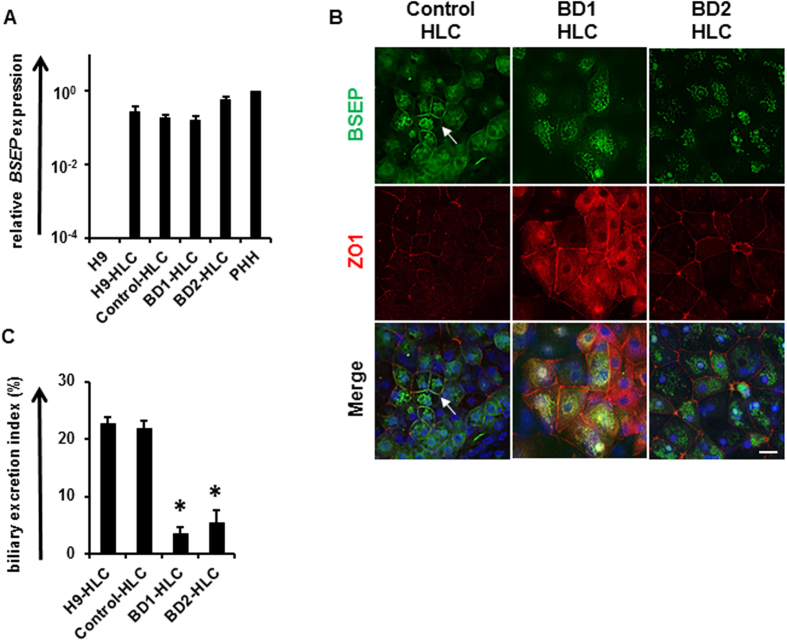
Analysis of BSEP expression and biliary excretion in the BD-HLCs. (**A**) Gene expression levels of *BSEP* in the HLCs as examined by real-time RT-PCR. The gene expression levels in PHH were taken as 1.0. Data represent mean ± SD (*n* = 3). (**B**) Immunofluorescence double staining of BSEP and ZO-1 in the different HLCs. White arrow indicates the co-localization of BSEP and ZO-1. (**C**) BEI of CLF-treated HLCs. Data represent mean ± SE (three independent differentiation experiments). Statistical significance was evaluated by one-way ANOVA in Tukey’s test. *P < 0.05. The scale bar represents 20 μm.

Next, we investigated whether the BD-HLCs could recapitulate the PFIC2 phenotype. The *BSEP* expression levels in the BD-HLCs were similar to those of Control-HLCs (Fig. 4A). In addition, *farnesoid X receptor (FXR*), which regulates the transcription of *BSEP,* was expressed in BD-HLCs (Fig. S3A). These results suggested that the expression level of *BSEP* transcripts in BD-HLCs was similar to that in Control-HLCs. To analyze the localization of BSEP in the HLCs, immunofluorescent double staining of BSEP and ZO-1 was performed, because ZO-1 is located on the canalicular membrane^29^. BSEP and ZO-1 were not co-localized in BD-HLCs, but were co-localized in the Control-HLCs (Fig. 4B, white arrow). This finding suggested that membrane BSEP was not expressed on BD-HLCs, which was also observed in the liver of the patients with BSEP deficiency (Fig. 1B, left and middle). It is also suggested that there might be a problem in BSEP trafficking in these two patients. Taken together, this data showed that BD-HLCs reproduced a pathological feature of PFIC2.

To investigate the capacity for bile acid uptake and synthesis in the BD-HLCs, the gene expression levels of the bile acid uptake transporter (Na^+^-taurocholate cotransporting polypeptide, *NTCP*) and bile acid synthesis enzyme (cytochrome P4507A1, *CYP7A1*) were examined by real-time RT-PCR. The gene expression levels of *NTCP* and *CYP7A1* were higher in the BD-HLCs than in PHHs (Fig. S3B,C). These results indicated that the BD-HLCs had the capacity to take up and synthesize bile acid. Next, to evaluate the biliary excretion capacity of HLCs, the biliary excretion index (BEI) was calculated using CLF^25^,^26^. The BEI values of the BD-HLCs were significantly lower than those of the Control-HLCs (Fig. 4C). Taken together, these results showed that the BD-HLCs could reproduce the pathophysiology of PFIC2, specifically the impairment of biliary excretion.

### Drug efficacy evaluation of 4PBA in the BD-HLCs

**Figure 5:**
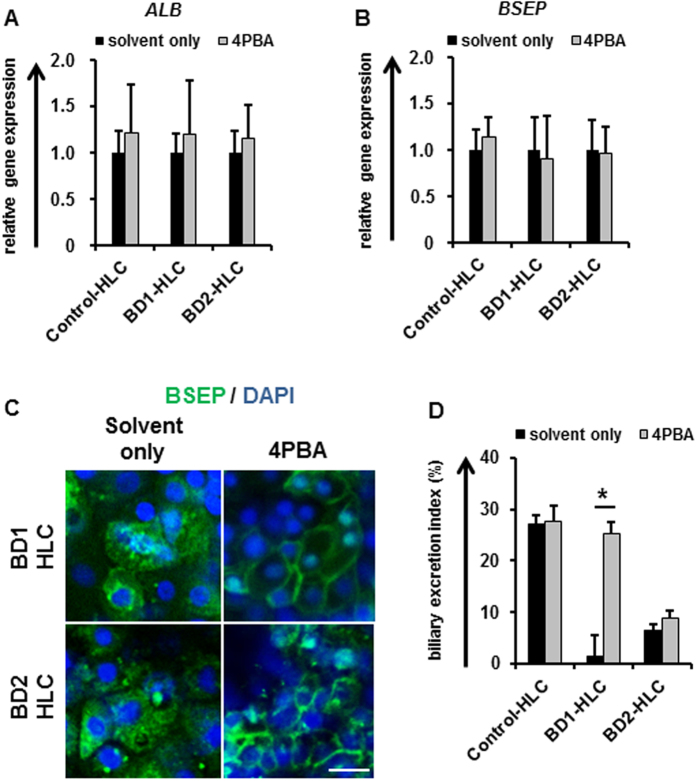
Analysis of the BD-HLCs treated with 4-phenylbutyrate. The BD1, BD2, and Control HLCs were treated with 1 mM 4PBA for 24 hours. (**A**,**B**) Gene expression levels of *ALB* and *BSEP* in the HLCs as examined by real-time RT-PCR. The gene expression levels in solvent-treated HLCs were taken as 1.0. Data represent mean ± SD (*n* = 3). (**C**) Immunofluorescence staining of BSEP in the HLCs. The scale bar represents 20 μm. (**D**) BEI of 4PBA-treated HLCs. Data represent mean ± SE (three independent differentiation experiments). Statistical significance was evaluated by Student’s t-test (*n* = 3). *P < 0.05.

Finally, to investigate whether BD-HLCs could demonstrate measurable responses to therapeutic agents, we examined the drug efficacy of 4PBA, a promising drug for treating patients with PFIC2^30^,^31^,^32^,^33^. It is known that 4PBA can improve membrane BSEP expression and biliary excretion capacity^30^. To investigate whether 4PBA treatment affected the BD-HLCs, the gene expression level of *ALB* was examined by real-time RT-PCR (Fig. 5A). No significant alteration of *ALB* gene expression levels was observed by 4PBA treatment, suggesting that 4PBA did not affect the hepatic function of the HLCs. Next, the *BSEP* gene expression level and membrane BSEP expression level in the 4PBA-treated HLCs were examined by real-time RT-PCR (Fig. 5B) and immunostaining (Fig. 5C), respectively. Although there was no significant alteration of *BSEP* expression level by this treatment, the membrane BSEP expression was increased by 4PBA treatment. These results suggested that 4PBA treatment did not promote transcriptional activation of BSEP, but altered other pathways, such as the pathway for membrane-protein trafficking and degradation^4^,^34^. In addition, the BEI was calculated to examine whether the biliary excretion capacity of the BD-HLCs could be rescued by 4PBA treatment. The BEI in BD1-HLCs treated with 4PBA was significantly higher than that in these cells treated with solvent only (Fig. 5D). On the other hand, 4PBA treatment did not increase the biliary excretion capacity of BD2-HLCs. This might be explained by assuming that the biliary excretion capacity of the mutated BSEP was very low that is in agreement with the previous bioinformatics analyses of the mutated BSEP function (Table S3), although the membrane BSEP expression levels were increased by 4PBA treatment. These results suggested that the drug efficacy could be evaluated by using BD-HLCs.

## Discussion

The aim of this study was to examine whether HLCs derived from patients with BSEP-deficiency could reproduce the disease characteristics of PFIC2. We show here that the pathophysiologic features of PFIC2, i.e., abnormality of BSEP expression and impairment of biliary excretion, were reproduced in BD-HLCs. To the best of our knowledge, this is the first case generating a cholestatic disease model, using iPSC technology^20^.

One-third of patients with PFIC are not diagnosed genetically^16^. Recently, mutations in tight junction protein 2 (*TJP2*) and *FXR* were found in patients with BSEP-deficiency, who harbored normal serum GGT and had no mutations in the *ABCB11* genes^15^,^35^. However, the gene or mutation responsible for PFIC has not been identified in the BD1 patient or other BSEP-deficient patients with normal serum GGT^10^. The combination of iPS technology and next-generation sequencing technology is expected to provide some clues^15^,^35^,^36^,^37^. Future studies may reveal the function of novel PFIC-related genes identified using next-generation sequencing analysis with patient-specific iPSC-derived HLCs.

In this study, we identified two novel compound heterozygous *ABCB11* mutations in c.-24C > A (5′-UTR) and c.2417G > A (p.G806D) and demonstrated that the 5′-UTR mutation resulted in aberrant splicing of mRNA followed by elimination of the proper initiation codon (Fig. 3A,B). The aberrantly spliced mRNA from the allele with this 5′-UTR mutation (c.-24C > A) does not appear to be translated into a functional BSEP protein. Some studies have shown that the *ABCB11* mRNA is aberrantly spliced in PFIC2^38^; however, the effects of mutations in the 5′-UTR of the *ABCB11* gene have not been reported previously. As the expression of the *ABCB11* gene is observed only in hepatocytes, the spliced form of *ABCB11* mRNA cannot be analyzed using other types of cells. It is difficult to obtain biopsy samples of the liver, although blood cells and dermal fibroblasts are often used to analyze genomic DNA or mRNA. Thus, we were able to analyze aberrant splicing of *ABCB11* gene using the HLCs instead of liver biopsy samples. The amino acid mutation (p.G806D) may also cause severe dysfunction of the BSEP protein as predicted by the Polyphen-2 and SIFT tools (Table S3). Accordingly, our data showed that the biliary excretion capacity of the BD2-HLCs was lower compared to Control-HLCs (Fig. 4C).

4PBA treatment promoted the membrane expression of BSEP (Fig. 5C). However, 4PBA treatment did not improve the capacity of bile secretion in BD2-HLCs (Fig. 5D). This discrepancy may be explained by the existence of mutation which causes defect in the transport activity. Consistently, Naoi *et al*. suggested that 4PBA may be an effective compound for patients with PFIC2 who retain transport activity of BSEP^31^. Because it is reported that CLF is also transported into bile canaliculi by not only BSEP but also other transporters^39^, it might be important to perform further analysis for evaluating BSEP function using the HLCs.

Our findings suggest a potential for dissecting PFIC2 pathogenesis at the cellular and molecular level in patient-specific HLCs. Our research also provides hope for discovering novel therapeutic options for treating BSEP deficiency and cholestasis in the future. In conclusion, this is the first report establishing a PFIC2 model in human hepatocytes, using iPSC technology. We believe that this model will promote further innovations in BSEP research.

## Additional Information

**How to cite this article:** Imagawa, K. *et al*. Generation of a bile salt export pump deficiency model using patient-specific induced pluripotent stem cell-derived hepatocyte-like cells. *Sci. Rep.* **7**, 41806; doi: 10.1038/srep41806 (2017).

**Publisher's note:** Springer Nature remains neutral with regard to jurisdictional claims in published maps and institutional affiliations.

## Supplementary Material

Supplementary Information
