# Supplementary material for: Generation of a bile salt export pump deficiency model using patient-specific induced pluripotent stem cell-derived hepatocyte-like cells
Source: Sci Rep. 2017 Feb 2;7:41806. doi: 10.1038/srep41806 (PMC5288783; doi:10.1038/srep41806)
Supplement: Supplementary Information [file srep41806-s1.pdf]

Supplementary Information

Generation of a bile salt export pump deficiency model using patient-specific induced pluripotent stem cell-derived hepatocyte-like cells

Authors

Kazuo Imagawa[1,2,3,†], Kazuo Takayama[2,3,4,†], Shigemi Isoyama[1], Ken Tanikawa[5], Masato Shinkai[6], Kazuo Harada[7], Masashi Tachibana[2], Fuminori Sakurai[2,8], Emiko Noguchi[9], Kazumasa Hirata[7], Masayoshi Kage[5], Kenji Kawabata[10,11], Ryo Sumazaki[1,*], Hiroyuki Mizuguchi[2,3,12,*]

Materials and methods

RNA isolation and reverse transcription-polymerase chain reaction (RT-PCR)

Total RNA was extracted from human ESC/iPSCs and their derivatives using ISOGEN (NIPPON GENE). The complemented DNA (cDNA) was synthesized using 500 ng of total RNA with a Superscript VILO cDNA synthesis kit (Thermo fisher scientific). Real-time RT-PCR was performed with SYBR Green PCR Master Mix (Applied Biosystems) using a StepOnePlus real-time PCR system (Applied Biosystems). Relative quantification was performed against a standard curve and the values were normalized against the input determined for the housekeeping gene, glyceraldehyde 3-phosphate dehydrogenase (*GAPDH*). The PCR primer sequences used in this study are described in **Table S1**. The *ABCB11* cDNA synthesized from the RNA of HLCs was amplified by RT-PCR using Ex Taq DNA polymerase. The primers 1F and 263R are listed in **Table S2**. The expected size of the PCR products was 263 bp for *ABCB11* Exon1-2-3-4. The PCR products were separated by electrophoresis on 2% agarose gels and visualized by staining with ethidium bromide **(Figure 3C)**.

Immunostaining

The cultured cells were fixed with 4% paraformaldehyde (PFA) in PBS for 20 min. After the incubation with 0.1% Triton X-100 (Wako) in PBS for 10 min, the cells were blocked with PBS containing 10% FBS and 1% bovine serum albumin for 1 hr, and incubated with a primary antibody at 4°C overnight. Finally, the cells were incubated with a secondary antibody at room temperature for 1 hr. The primary and secondary antibodies used in this study are described in **Table S4**, respectively. The paraffin section was obtained from the liver and fixed with formalin. The liver section was stained for BSEP and MRP2 by using DAB immunohistochemistry.

Transmission electron microscopy

The samples were fixed with 2% paraformaldehyde and 2% glutaraldehyde (GA) in 100 mM phosphate buffer (PB) pH 7.4 at incubation temperature, and then they were placed in a refrigerator for 30 min in order to lower the temperature to 4°C. Thereafter, they were fixed with 2% GA in 100 mM PB at 4°C overnight. After three washes with 100 mM PB, the samples were postfixed with 2% osmium tetroxide in 100 mM PB at 4°C for 1 hr. They were then dehydrated in graded ethanol solutions (50%, 70%, 90%, 100%). The samples were transferred to a resin (Quetol-812; Nisshin EM Co.), and were polymerized at 60°C for 48 hr. Ultrathin sections were stained with 2% uranyl

acetate at room temperature for 15 min, then washed in distilled water and secondarily stained with Lead stain solution (Sigma-Aldrich Co.) at room temperature for 3 min. The grids were observed by a transmission electron microscope (JEM-1400plus; JEOL, Ltd.) at 80 kV. Images were taken with a CCD camera (VELETA; Olympus Soft Imaging Solutions GmbH).

Enzyme-linked immuno-sorbent assay (ELISA)

The culture supernatants, which were incubated for 24 hr after fresh medium was added, were collected and analyzed for the amount of albumin (ALB) secretion by ELISA. Human Albumin ELISA Quantitation Set was purchased from Bethyl Laboratories. ELISA was performed according to the manufacturer’s instructions. The amount of ALB secretion was calculated according to each standard followed by normalization to the protein content per well.

Biliary excretion and bile canaliculi formation assay

On day 25 of the hepatocyte differentiation, the Control-HLCs were treated with 5 µM cholyl-lysyl-fluorescein (CLF) (Corning) for 30 min. The cells were then washed with HBSS buffer, and observed with a fluorescence microscope **(Figure S2)**.

Embryoid body formation and spontaneous differentiation *in vitro*

To form the embryoid bodies (EBs), human ESCs/iPSCs were cultured with DMEM (Thermo fisher scientific) supplemented with 10% FBS, 120 µg/mL streptomycin, and 200 µg/mL penicillin on low-attachment 96-wel plates for 4 days. The EBs were then plated onto gelatin-coated dishes, and cultured with the same culture medium for 10 days. The culture medium was changed every other day.

Treatment with 4-phenylbutyrate

The HLCs were treated with 1 mM 4-phenylbutyrate (4PBA; Sigma) for 24 hours.

Table S1. List of primers used for RT-PCR in this study

Genes	Forward primer	Reverse primer
OCT4	GGTTCTCGATACTGGTTCGC	GTGGAGGAAGCTGACAACAA
NANOG	AGAAGGCCTCAGCACCTAC	GGCCTGATTGTTCCAGGATT
AFP	TGGGACCCGAACTTTCCA	GGCCACATCCAGGACTAGTTTC
TTR	TCATCGTCTGCTCCTCCTCT	AGGTGTCATCAGCAGCCTTT
NKX2.5	CTGTCTTCTCCAGCTCCACC	TTCTATCCACGTGCCTACAGC
BRACHYURY	TATGAGCCTCGAATCCACATAGT	CCTCGTTCTGATAAGCAGTCAC
NEUROD1	ATCAGCCCACTCTCGCTGTA	GCCCCAGGGTTATGAGACTAT
PAX6	TGGGCAGGTATTACGAGACTG	ACTCCCGCTTATACTGGGCTA
ALB	GCACAGAATCCTTGGTGAACAG	ATGGAAGGTGAATGTTTTCAGCA
BSEP	CCAGGAAAAGCATGTGTGAA	CGACAGCAATTCCAGCATAG
FXR	AACTGAGGTAGCAGAGATGC	GCTCTGTCTCCACAAACAAC
NTCP	AGAAGGTGGAGCAGGTGGT	ATCTTGGTCTGTGGCTGCTC
CYP7A1	GAGAAGGCAAACGGGTGAAC	GCACAACACCTTATGGTATGACA
SeV	GGATCACTAGGTGATATCGAGC	ACCAGACAAGAGTTTAAGAGATATGTATC

Table S2. List of primers used for *ABCB11* sequence analyses in this study

Primer name	sequence
1F	GAATGATGAAAACCGAGG
119F	CAATTACCATGTCTGACTCAGT
263R	CCAACTCTAACGCCATCAC
509F	GTTGTGGGTTGCTGAACATC
701F	GCAATTCAGTGGGGGAGCTG
983F	CATCAATGAGAACAGTGGCTGC
1514F	CAGCACTGCAACTCATTCAG
2014F	GAACATGGCACTGCAGTGG
2316F	TCCTGTGCAGGAAGAAGTTG
2330R	TCTTCCTGCACAGGAATGTC
2508F	GATCAATGGTGTGTGCCTAC
2520R	CACACCATTGATCTGTGACC
3011F	CACTTGAGACTGAGCTGGAG
3565F	GTAAATGTCCAGTTCCTCCGC
4104R	TCTTGCATTGGGTCAACTGAT
Exon2F	GACTGTGGCTTATCTTTCC
Exon2R	GGTGTGTTCTTACCTAGTGCTTTC
Exon20F	AGATCCACAGCTTACATTAG
Exon20R	CTGAGGTCATCAAACCAGGC

Table S3. Prediction of pathogenicity of the *ABCB11* mutation

ABCB11	exon	location	protein	SIFT	PolyPhen-2
c.2417G>A	20	TM8	G806D	0<br>damaging	0.999<br>probably damaging

TM: transmembrane

SIFT: The score less than 0.05 indicates that the amino acid substitution is predicted as damaging.

PolyPhen-2: The score ranges from 0.000 (most probably benign) to 0.999 (most probably damaging).

Table S4. Antibodies used in this study

Antigen	Type/ Fluorescent Dye	Company
ALB	goat	Bethyl Laboratories
BSEP	mouse	Santa Cruz Biotechnology
ZO-1	rabbit	Thermo Fisher Scientific
OCT3/4	mouse	Santa Cruz Biotechnology
NANOG	mouse	Santa Cruz Biotechnology
AFP	mouse	Santa Cruz Biotechnology
cTnT	mouse	Thermo Fisher Scientific
Nestin	rabbit	Sigma
mouse	alexa fluor 488	Molecular Probes
rabbit	alexa fluor 488	Molecular Probes
goat	alexa fluor 488	Molecular Probes
rabbit	alexa fluor 594	Molecular Probes

Figure S1

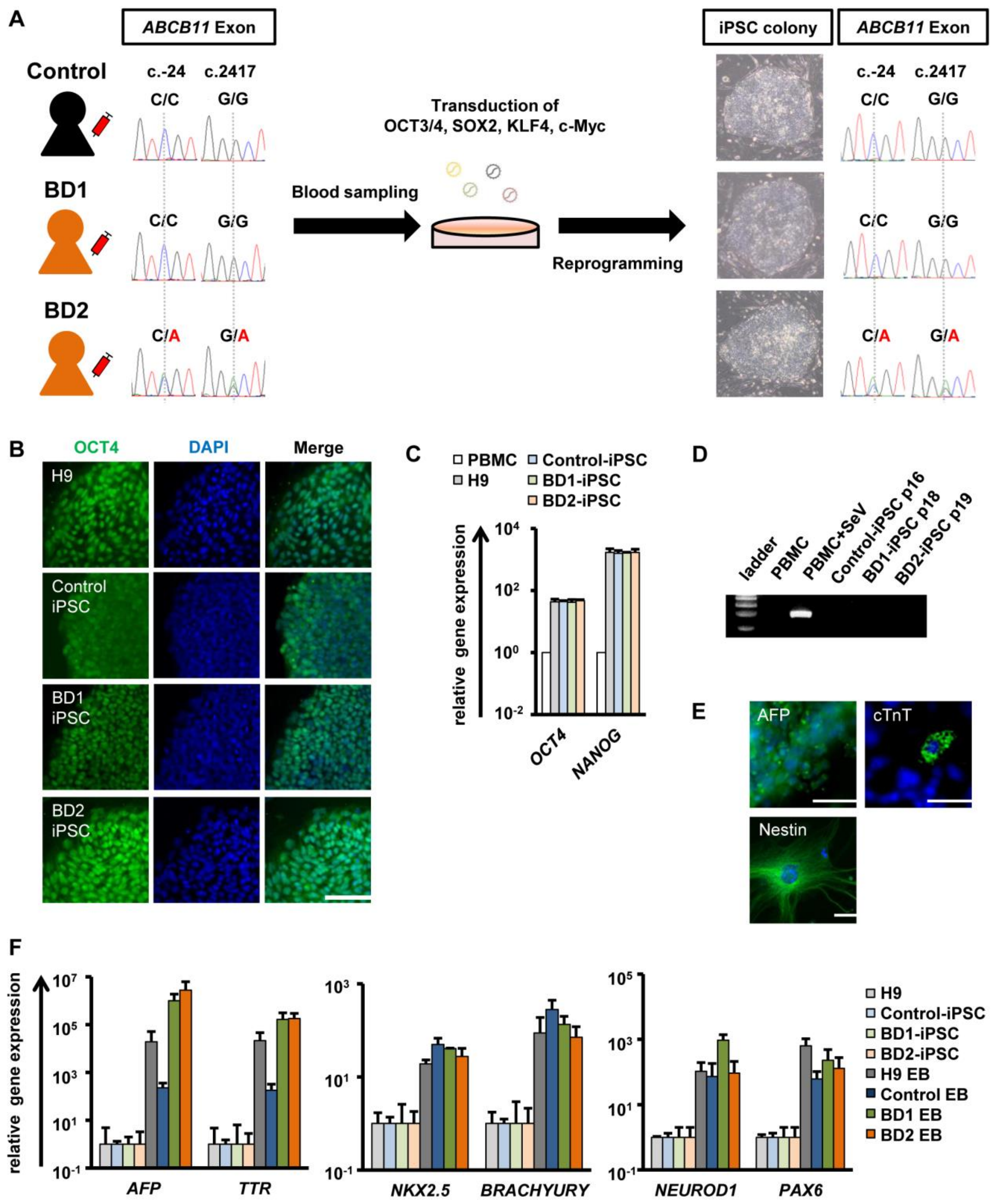

Figure S1. Generation of human iPSCs from PBMCs of a patient with BSEP deficiency

(A) Human iPSCs were generated from the PBMCs of the healthy control and two patients with BD by using Yamanaka four factor-expressing SeV vectors. The

morphology of the human iPSC colony is shown. The sequence of the *ABCB11* gene in the DNA extracted from the three human iPSC lines was also analyzed. (B) The expression of the pluripotent marker, OCT4, in the iPSCs, and in human ESCs (H9) was examined by immunostaining analysis. Nuclei were counterstained with DAPI. The scale bar represents 40 μm. (C) The gene expression levels of pluripotent markers (*OCT4* and *NANOG*) in the PBMCs, H9, and iPSCs were examined by real-time RT-PCR. The gene expression levels in the PBMCs were taken as 1.0. Data represent the mean ± SD (*n*=3). (D) RT-PCR analysis for the SeV genome was performed by using RNA extracted from the PBMCs, SeV-infected PBMCs, and individual human iPSCs passaged more than ten times. The amplicon was subjected to 2% agarose gel electrophoresis. (E) The expressions of AFP (an endodermal marker), cTnT (a mesodermal marker), and Nestin (an ectodermal marker) in the EBs were examined by immunostaining analysis. Nuclei were counterstained with DAPI. The scale bar represents 40 μm. (F) The gene expression levels of the endodermal markers (*AFP* and *TTR*), mesodermal markers (*NKX2.5* and *BRACHYURY*), and ectodermal markers (*NEUROD1* and *PAX6*) in the EBs were examined by real-time RT-PCR. The gene expression levels in the undifferentiated cells were taken as 1.0. Data represent the mean ± SD (*n*=3).

Figure S2

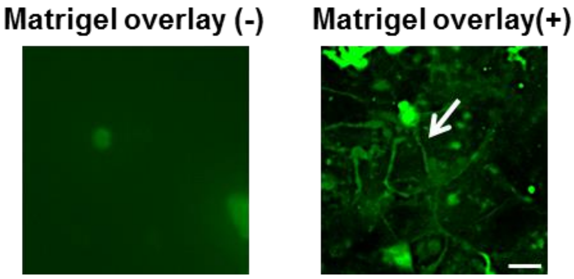

Figure S2. Characteristics of the hepatocyte-like cells

CLF accumulation in bile canaliculi was not observed in the Control-HLCs which were not overlaid with Matrigel (left panel), but it was detected in the Control-HLCs overlaid with Matrigel (right panel). The white arrow indicates bile canaliculi between the HLCs. The scale bar represents 20 μm.

Figure S3

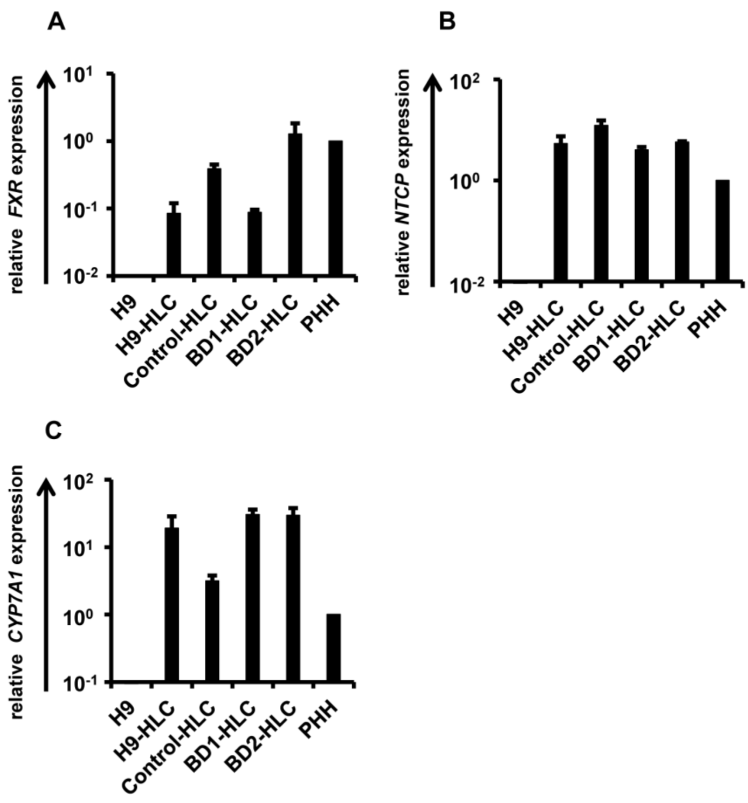

Figure S3. Gene expression levels of FXR, biliary uptake transporter and bile synthesis

(A, B) The gene expression levels of *FXR* (A), *NTCP* and *CYP7A1* (B) were examined by real-time RT-PCR. The gene expression level in PHH was taken as 1.0. Data represent the mean ± SD (*n*=3).
